# A collaborative, mixed‐methods evaluation of a low‐cost, handheld 3D imaging system for child anthropometry

**DOI:** 10.1111/mcn.12686

**Published:** 2018-10-18

**Authors:** Joel Conkle, Kate Keirsey, Ashton Hughes, Jennifer Breiman, Usha Ramakrishnan, Parminder S. Suchdev, Reynaldo Martorell

**Affiliations:** ^1^ Doctoral Program in Nutrition and Health Sciences Laney Graduate School, Emory University Atlanta Georgia; ^2^ Hubert Department of Global Health, Rollins School of Public Health Emory University Atlanta Georgia; ^3^ Division of Nutrition, Physical Activity and Obesity National Center for Chronic Disease Prevention and Health Promotion. U.S. Centers for Disease Control and Prevention Atlanta Georgia; ^4^ Department of Pediatrics, School of Medicine Emory University Atlanta Georgia

**Keywords:** 3D, anthropometry, height, length, nutritional status, user experience

## Abstract

3D imaging for body measurements is regularly used for design of garments and ergonomic products. The development of low‐cost 3D scanners provided an opportunity to extend the use of 3D imaging to the health sector. We developed and tested the AutoAnthro System, the first mobile, low‐cost, full‐body, 3D imaging system designed specifically for child anthropometry. This study evaluated the efficiency, invasiveness, and user experience of the AutoAnthro System. We used a mixed‐methods, collaborative approach that included a quantitative time‐motion study and qualitative interviews of anthropometrists. For cooperative children, anthropometrists considered the use of 3D imaging an easy, “streamlined experience,” but with uncooperative children, anthropometrists reported that capturing a good quality scan was out of their control. The mean time to complete a full set of scans was 68 s (standard deviation [SD] 29), compared with 135 s (SD 22) for a set of manual measurements (stature, head circumference, and arm circumference). We observed that crying was more common during manual measurement, and anthropometrist interviews confirmed that 3D imaging was less stressful for children than manual measurement. In a previous publication, we showed the potential of 3D imaging to produce reliable and accurate measurements. In this study, we found that anthropometrists were not ready to abandon manual equipment for 3D scanners because of difficulty in measuring uncooperative children. Revising the AutoAnthro System to address anthropometrists' concerns on capturing good quality scans of uncooperative children should help to facilitate widespread use of 3D imaging for child anthropometry.

**Key messages**

- For most children, 3D imaging was an efficient and non‐invasive way to capture anthropometric data, but further software development is needed before recommending AutoAnthro for regular nutritional assessment.
- Additional research that includes a qualitative component is needed to replicate our findings in a household or community setting and in a context where people are not familiar with the technology.
- The value of 3D imaging for anthropometry may extend beyond efficiency gains and quality improvement. 3D imaging may enable future development of novel anthropometric indicators that are not feasible with manual measurement and are better predictors of outcomes of interest.

## 1 INTRODUCTION

3D imaging for anthropometry was developed in 1989 for use in the garment industry, which relied on sizing surveys for pattern development (Jones, West, Harris, & Read, 1989). By the late 1990s, a large‐scale sizing survey provided scan‐derived anthropometric data to manufacturers for design of garments and ergonomic products (Robinette, Daanen, & Paquet, 1999). 3D scanners are now commonplace in national sizing surveys, with multiple countries adopting the technology (Charoensiriwath, 2008; Kim, You, & Kim, 2017; Wells, Treleaven, & Cole, 2007). 3D imaging has also been used for anthropometry in the health sector. Over the last decade, multiple studies tested various 3D scanners for measurements of clinical interest, such as adult height (Kuehnapfel, Ahnert, Loeffler, Broda, & Scholz, 2016), waist/hip circumference (Jaeschke, Steinbrecher, & Pischon, 2015; Pepper et al., 2010), body fat (Garlie, Obusek, Corner, & Zambraski, 2010; Wang et al., 2006), body surface and volume (Adler et al., 2017; Barnes, 2010; Wells et al., 2007), and body shape (Loffler‐Wirth et al., 2016). Research on metabolic syndrome used scan‐derived anthropometry (Jaeschke et al., 2015; Lin, Chiou, Weng, Fang, & Liu, 2004), and over the last few years, large‐scale epidemiological studies of adults used 3D scanners (Kuehnapfel, Ahnert, Loeffler, & Scholz, 2017; Loffler‐Wirth et al., 2016). However, previous research on 3D imaging for anthropometry used expensive, stationary scanners; 3D imaging is not yet used in health and nutrition surveys, and its use in health facilities is limited to research and specialized purposes, such as cranial remoulding orthoses (Weathers et al., 2014).

The development of “light‐coding” technology reduced the cost and size of 3D scanners and led to use in the gaming industry; and in 2013, a Kickstarter campaign funded the development of Structure Sensor (Occipital, San Francisco, CA), an open‐source 3D scanner that attaches to a tablet or phone. The development of low‐cost, mobile, open‐source scanners provided an opportunity to extend the use of 3D imaging to common uses of anthropometry in the health sector, such as nutritional screening and surveillance. We tested the AutoAnthro System, a tablet‐based 3D imaging system for anthropometry designed for children under 5 years of age. We were particularly interested in a system designed for young children because basic measurements taken during surveys or in healthcare settings, such as length and circumferences, are often of poor quality in this age group (Assaf, Kothari, & Pullum, 2015; Corsi, Perkins, & Subramanian, 2017; Lipman et al., 2004; Pullum, 2008; Yin, Dai, Li, Xie, & Ren, 2013), and there were recent global calls from the United Nations Children's Fund and the United States Agency for International Development for technology to improve child anthropometric data quality (UNICEF Supply DIvision Innovation Unit, 2017; USAID, 2016). In a previous publication, we showed that the AutoAnthro System produced measurements of child length, height, head circumference, and arm circumference that were as reliable as expert manual measurement and concluded that after minor software adjustments to remove systematic inaccuracy, the imaging system could produce measurements that were the same quality as the current gold standard (Conkle et al., 2018). This study evaluated the efficiency, invasiveness, and user experience of the newly developed 3D imaging system. The purposes of our research were to inform further development of AutoAnthro and to go beyond measurement quality to assess the potential for widespread use of 3D imaging for nutritional assessment of children in the health sector.

## 2 MATERIALS AND METHODS

The Body Imaging for Nutritional Assessment Study (BINA) compared traditional, manual anthropometry to 3D imaging and was approved by the Emory Institutional Review Board. For BINA, we used AutoAnthro (BST, Atlanta, GA), a custom software developed by Body Surface Translations for capturing and processing scans using the Structure Sensor. We calibrated AutoAnthro to produce length, height, head circumference, and arm circumference based on a sample of 36 children who were scanned and measured manually. We focused on stature because it is regularly used in surveys and health facilities to assess nutritional status and on head and arm circumference because they are regularly used in healthcare settings for screening of undernutrition and abnormal development, respectively. Following calibration, we carried out a cross‐sectional, validation study on 474 apparently healthy children under 5 years of age. Five trained anthropometrists conducted all measurements and scans in day cares and medical facilities in Atlanta, USA. For manual measurement, we followed measurement protocol used by the World Health Organization for development of the 2006 World Health Organization Growth Standards (de Onis, Onyango, Van den Broeck, Chumlea, & Martorell, 2004). For scanning, we developed a new protocol. We scanned children over 2 years of age standing up with their arms in three specified poses and children under 2 years of age lying down with their arms extended away from the torso. Detailed methodology for BINA and the sample characteristics are available in previous publications (Conkle, 2017b; Conkle, Ramakrishnan, Flores‐Ayala, Suchdev, & Martorell, 2017; Conkle et al., 2018), and both the study data and the study manual, which include detailed scanning protocol, are available via Open Science Framework (Conkle, 2017a). This paper presents quantitative and qualitative research on the experience of using 3D scanners for anthropometry. We conducted a time‐motion study and qualitative interviews of anthropometrists, adopting a mixed‐methods approach to provide a comprehensive assessment of experience. We collected data at the end of the BINA validation study (February 2017) to maximize anthropometrists' experience with AutoAnthro. We worked with anthropometrists to improve the relevance and application of our research (Israel et al., 2005), referring to Evidence‐based Principles to Guide Collaborative Approaches to Evaluation (Shulha, Whitmore, Cousins, Gilbert, & Al Hudib, 2015) to inform our collaborative approach. To illustrate the collaborative nature of this research, it is important to note that BINA anthropometrists helped to design the research, developed tools, reviewed and revised manuscript drafts, and approved the final manuscript.

### 2.1 Time‐motion study

The study compared time required to take manual measurements versus 3D scanning using continuous observation based on milestone timing (Lopetegui et al., 2014). We followed Suggested Time and Motion Procedures developed by Zheng, Guo, and Hanauer (2011), and we developed and pretested the study protocol and tools. The lead researcher and one BINA anthropometrist defined all measurement tasks and developed cues for start/stop times based on those tasks. A single observer recorded time using a stopwatch in order to improve reliability. We set the minimum sample size at 22 children on the basis of achieving 90% power to detect a 30‐s difference between measurement methods (Paired *t*‐test, *α* < 0.05), which we considered a meaningful difference for efficiency of nutritional screening. The time‐motion study sample was a subsample of children who were measured in BINA, and we used purposeful selection to ensure that approximately one half of the subsample was under 2 years of age.

We did not include the time to establish rapport or undressing because these activities were required for both scans and manual measurement. The protocol for manual measurement required undressing to undergarments, whereas the scan‐based measurements required that the child be undressed to undergarments or to skin‐tight leotard/shorts. We also did not measure the time required to set up equipment but did include data entry in manual measurement time. Each child was scanned and measured twice by two different measurers, resulting in four sets of scans and four sets of manual measurements. Children were scanned first and then immediately measured manually. A set of scans consisted of six 1‐s scans, and a set of manual measurements included length or height, head circumference, and arm circumference. Weight was not included in timing of manual measurements because we did not calculate weight from scans. To compare scans and manual measurements, we timed the four sets as one unit, which simplified measurement by reducing the number of cues. For reporting, we divided the total time by four so that the average time presented in this study represents the time required for a single set of scans or manual measurements. To determine the time required for each manual measurement type (stature, HC, and MUAC), we observed a single measurer and took the mean from their two observations. The time reported for each measurement type represents a single measurement. We paused timing of measurements when measurers were interrupted. If the interruption was not related to the child's behaviour, we did not record the length of the interruption. In the case that a child became too upset to continue measuring and the measurer had to stop measuring to calm the child, we timed the interruption and noted if it occurred during scans or manual measurements. Timing interruptions allowed us to calculate measurement time with and without interruptions; the two estimates were needed because pausing measurements may have been more common in BINA than in a household survey because of the facility setting and the absence of the primary caregiver. We did not pause timing for technical difficulties with the 3D scanner. While timing measurements, we also observed if a child cried and noted if crying occurred during scans, manual measurements, or both. We used SPSS 20 (IBM Corp., Armonk, NY) for data analysis.

### 2.2 Interviews

We used grounded theory from a constructivist point of view for qualitative design and analysis (Charmaz, 2006). In a constructivist approach, it is acknowledged that the research findings come through the view of the researcher, and it is important to consider the characteristics of the researcher, which we briefly do here. The first author (J. C.) led the qualitative component of the study and had relevant training in the methods used in this study. He also supported training of anthropometrists and supervised data collection for the main BINA study. Before carrying out interviews, the lead researcher had already developed opinions on the new technology through experience with BINA; every effort was made to develop a joint understanding of issues with interviewees and to not impose pre‐existing ideas on to interviewees.

We sought to include everyone with extensive experience using the AutoAnthro System, which limited the intended sample to the five BINA anthropometrists, who all agreed to participate. In consultation with anthropometrists, we first conducted the written, in‐depth interviews (IDI) and followed IDIs with a focus group discussion (FGD) that was facilitated by the lead researcher. One of the anthropometrists and the lead researcher developed a questionnaire with open‐ended and probing questions on the identified categories of efficiency, invasiveness, and the general user experience, with the latter category broadly covering the advantages and disadvantages of 3D imaging in comparison with manual measurements. The lead researcher and one anthropometrist independently coded IDIs line‐by‐line, and the lead researcher created code families and memos on the basis of both sets of coded IDIs. We used ATLAS.ti 7 (Scientific Software Development GmbH, Berlin, Germany) for analysis.

Following the analysis of all individual, written interviews, we designed a semistructured interview guide with open‐ended questions for the FGD. The primary purpose of the FGD was to clarify and expand on points raised in the written responses. The lead researcher facilitated the FGD, which was held in a private conference room at Emory University. The proceedings were audio‐recorded with a mobile phone and transcribed with Dragon NaturallySpeaking (Nuance, Burlington, MA). The lead researcher coded the FGD line‐by‐line and revised code families generated from IDIs to incorporate the new information from the FGD. The lead researcher created a network map of code families to facilitate further memoing and identified theories from the data. As authors, anthropometrists reviewed and revised findings, which functioned as a “member check” to enhance trustworthiness of findings. We referred to the 2014 Standards for Reporting Qualitative Research from O'Brien (O'Brien, Harris, Beckman, Reed, & Cook, 2014) to report qualitative findings.

## 3 RESULTS

### 3.1 Time‐motion study

**Figure 1:**
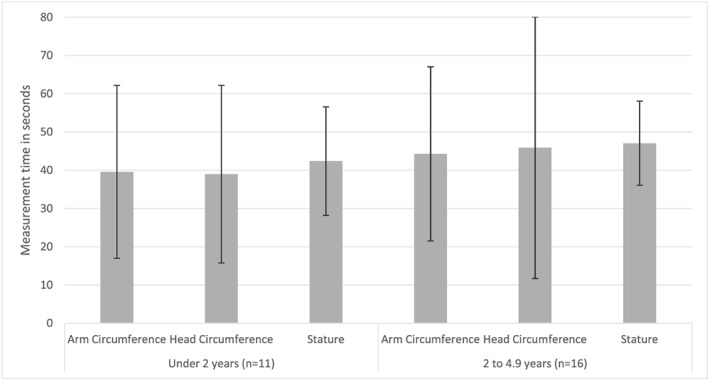
Mean measurement time for manual measurements. Measurement time taken from the first measurer average for each child. Time required for a single measurement (bars); 95% confidence interval represented by line with caps

We observed and recorded measurement time for 27 children under 5 years of age. On average, it took just over a minute (68 s) to complete a set of scans compared with over 2 min (135 s) for a set of manual measurements. The differences in measurement time between scans and manual measurement were statistically significant but did not differ by age group (Table 1). At the individual level, manual measurements took longer to complete for all children except one. For the child whose scans took longer, the scanner was malfunctioning. There was little difference between stature, HC, and MUAC for measurement time, with each measurement taking close to 40 s (Figure 1). Differences remained small after disaggregating by age group. For children under 2 years, the time for the various manual measurements ranged from 39 to 42 s and for children over 2 years from 44 to 47 s.

**Table 1:** Difference in the time required to complete one set of scans and manual measurements, BINA 2017

		Time to complete measurements, mean (*SD*), s		Mean difference, s	
Age group, year	*n*	Scanning	Manual	Mean (95% CI)	Significance^a^
Under five	27	68 (29)	135 (22)	−67 (−80, −54)	<0.001
Under two	11	63 (23)	121 (20)	−58 (−80, −35)	<0.001
2–4.9	16	71 (32)	144 (18)	−73 (−91, −56)	<0.001

*Note*. BINA: Body Imaging for Nutritional Assessment Study; CI: confidence interval; SD: standard deviation.

a Paired samples *t*‐test.

Around one fifth of children cried during manual measurements, and all of them were under 2 years of age (Table 2). Only one child cried during scans (4%), and that child also cried during manual measurements. Measuring was interrupted by the child's behaviour on two occasions; both interruptions occurred during manual measurements, and the average time of the interruption was 43 s. Including interruptions increased average manual measurement time by approximately 1 s, increasing mean measurement time to 136 s.

**Table 2:** Crying episodes and interruptions caused by noncompliance during scans and manual measurement, BINA 2017

Age, year	*n*	Number of children crying			Interruptions	
		Scans or manual	Scans	Manual	Number^a^	Average time, s
All	27	6	1	6	2	43
Under 2	11	6	1	6	2	43
2 to 5	16	0	0	0	0	n/a^b^

*Note*. BINA: Body Imaging for Nutritional Assessment Study.

a Both interruptions occurred during manual measurement.

b Not applicable because no interruptions in this age group.

### 3.2 Interviews

We completed five IDIs and one FGD, with participation in both from all five anthropometrists who had extensive experience using the AutoAnthro System. The five anthropometrists were all women with postsecondary degrees. After merging similar codes, we identified 96 action‐oriented codes and 15 code families: time, cooperation, ease of use, staff, learning, invasiveness, caregiver, individual, child's age, clothing, experience, touch, safety, environment, and dependability. Table 3 presents the code families and selected and associated codes and quotations. We identified two core themes, or theories, from the data that related to favourable and unfavourable perceptions of using the AutoAnthro System: “streamlined experience” and “quality control.”

**Table 3:** Results of coding and memoing of anthropometrists' interviews, BINA 2017

Code family	Selected codes (underlined) and selected quotations	Summary of memos (code families in bold)
Time	Cooperation same for both: “Generally, if a mild mannered child is cooperative for one set of measures they will be for the other, but the 3D scanning takes LESS TIME than the physical.” Child moving: “There are “sweet spots” in terms of age that makes the scanning faster than traditional measurements. Newborns and young infants … can be scanned faster …”	A major driver of the time required to complete measurements was child **cooperation**, which itself was driven by the **age** and temperament of the child, and **invasiveness** of the measurement method. Most anthropometrists reported that scanning took less time than manual measurements when children were cooperative. All anthropometrists reported that scanning took more time for uncooperative children compared with cooperative children, and some felt that scans took more time than manual measurements for children 6 months to 3 years of age.
Cooperation	Crying: “… reactions during physical measurements occurred while children under two were getting their length measurement taken. Most children, even the most compliant, did not enjoy the length board and usually cried, screamed, or tried to stand up.” Assessing blame for no cooperation: “… we just did not start anything on them because they were so uncooperative, so I would not blame that on the scans or the physical measurements. I would kind of blame it on the whole process. Distracting child: “Except for height/length, there is not an exact [full body] pose … for physical measurements. The measurer can move around the child to do the measurements. It is easier to distract a child during the physical measurement process. They can watch a video or play a game on the iPad …”	The method of measuring, scanning versus manual, was not the main determinant of cooperation. However, length was consistently reported to be particularly difficult. Anthropometrists viewed the child's temperament as important and viewed **child's age** as the best predictor of cooperation. Anthropometrists reported that removal of **clothing** and “stranger anxiety” could initially cause distress of the child, leading to poor cooperation and refusal before attempting measurement in some cases. Some anthropometrists reported that once measurement began, refusals occurred exclusively during scanning, whereas others reported that refusals only occurred during manual measures. During measurement, distraction was the main strategy used by anthropometrists to foster cooperation, and there was consensus that showing videos was the best distraction tool. Scanning uncooperative children was especially challenging because the child was unable to move, anthropometrists could not **touch** the child, and distraction was more difficult.
Ease of use	Easy to carry: “One of the major benefits … is that the device is extremely light, can fit in a small bag, and is very easy to operate. This is unlike the manual equipment, which is heavy and cumbersome during transportation.” Using both tools: “I think that overall, the scanning technology is easier/faster/more convenient for children of all ages. If I were tasked with measuring children with either tool, I would want to have the scanning technology as my primary method, and have the traditional tools as a backup for cases where it wasn't feasible.” Child moving: “I would say 3D scanning is more difficult, slower, and less dependable with an uncooperative/misbehaving child. Since we can't touch them while getting a scan, they can run or move around, making it impossible to capture a good scan.”	Anthropometrists commented that the physical characteristics of the scanner, small and lightweight, made it easy to use. **Learning** to use the scanner was easy according to anthropometrists, who also commented that the scanning equipment was sturdy and did not require sanitization. Anthropometrists did not consider charging the scanner battery to be a big burden but did report that forgetting to charge led to data collection delays on occasion. There was no reported potential for harm to the child from the scanners, but anthropometrists reported needing to explain the **safety** of scanning technology to caregivers. Children's previous **experience** with cameras facilitated easy use of scanners, but the requirement to remove **clothing** was a burden. Most anthropometrists reported that completing all required scans was generally easy, but that some “trial and error” was necessary and that scanning became difficult in specific circumstances. **Environment**, specifically lighting (attributed to both natural and fluorescent light), affected scanner functionality and made data collection more difficult. The biggest reported challenge to use the scanners was getting an uncooperative child to stay in position long enough to obtain adequate scans without being able to **touch** the child.
Staff	Not needing trained staff: “I think it's helpful to have two staff for manual measurements. It's helpful for scans to have a staffer and someone else to position the kid. It does not necessarily have to be someone who is trained.” Seeing different parts: “I think both always need two if you want to be accurate. For height and length someone has to be watching one end of the body. If you are doing scans, unless you have a perfect kid who was understanding your verbal directions …, [a single operator] would have to walk over change their arms, come back scan.”	Reported staff needs varied from one to three depending on the measuring method and **age** of child. For uncooperative children under 2 years of age anthropometrists reported needing three people to get an accurate length measurement. For manual measures at least two trained staff were necessary because height measurement requires simultaneous viewing of different parts of the body to ensure correct positioning. For scanning there was some agreement that an assistant was needed for most children and helpful for all children, but the assistant did not necessarily need to be formally trained. Some anthropometrists reported that with a cooperative child who followed instructions some manual measurements and scanning could be completed by a single measurer. For both manual measures and scanning anthropometrists viewed the use of an assistant as important for reducing measurement **time**.
Learning	Working by trial and error: “Learning to use 3D imaging was relatively easy. Besides having to adjust the box on the screen to fit the object/person … it is just like taking a picture. Most of the lessons were learned through trial and error …” Being confident in measurement: “Instructions on using the 3D imaging was pretty straightforward … I'm not sure if all of the measurers' questions concerning what constitutes a ‘good scan’ were ever completely answered. Though it was pretty obvious on what signified a ‘bad scan’.”	There was unanimous agreement that 3D imaging was easy to learn; it was like taking a picture. The custom software for scanning did not require much user input. However, trial and error was necessary during data collection to learn how to deal with various circumstances. For example, anthropometrists found that it was not possible to scan in hallways or to use two scanners at the same time on a single child. For the most part anthropometrists learned how to ensure that **environment** did not affect scans. However, at the end of data collection anthropometrists still did not feel that they could recognize a “good scan,” and they could not perfectly predict when lighting would affect scans.
Invasiveness	Receiving medical care: Children generally detest getting their recumbent length taken. If they are old enough, they might think they are getting a shot when we do MUAC, even when we explain what we're doing. They often think whatever physical measurements we are going to do will hurt – Because they associate us with medical professionals. They tend to not have these fears with scans. Being scared: For traditional measuring a lot of children first tend to be a little afraid … because our process is very similar to what they experience when they visit the doctor's office and they associate going to the doctor with getting painful shots. After seeing that what we are doing with them is not painful, I've noticed that most children are pretty relaxed and happy to be measured. For 3D imaging, children seem to have more fun and are excited to do the poses (sometimes too excited). Because it's kind of like taking a photograph and there is way less touching involved, I think most children are way more comfortable with this method.	Anthropometrists defined measuring invasiveness as causing the child to be “uncomfortable,” “anxious,” or “distressed;” and reported related behaviours of “crying,” “screaming,” or “moving away.” Removal of **clothing** was seen as invasive for some older children, and this was related to “stranger anxiety.” Nearly all anthropometrists reported that children were more comfortable with scanning because they were used to having a picture taken. Previous **experience** also affected manual measurement; all anthropometrists reported that children related manual measurements to a doctor's visit, with MUAC being related to getting shots. All anthropometrists reported that length caused the most distress, and the sense of confinement was cited by some as the underlying reason. **Touch**ing occurs for manual measurements and in some cases children were anxious about being touched, while for others touching was a source of comfort. Anthropometrists also considered the caregiver's reaction when considering invasiveness. Anthropometrists reported that caregivers may be more comfortable with manual measures because they are already familiar with them, and because there is an aversion to taking what appears to be a picture when the child is undressed. However, negative reaction from **caregivers** was reported for the length measurement.
Caregiver	Parents make harder: “I noticed that children tend to respond negatively to any of the measures when there was a parent around.” Discomfort with stranger: Many times, the child/infant did not like being touched by strangers (us). The whole process generally went better when we had a caregiver assist. Feeling awkward: “… showing a scan to the caregiver was a way to reassure them that the 3D image was much different than a photograph and that the child's identity and privacy was protected.”	Multiple anthropometrists felt that the presence of a caregiver made measurement more difficult, but all agreed that undressing the child was easier with a caregiver present. Some anthropometrists reported that uncooperative behaviour of the child during measurement was more common when a parent was present. Some anthropometrists felt compelled to show caregivers the scan of the child to reassure them that it was not an identifiable photograph. Anthropometrists reported that caregivers expressed that previous manual measures of their child in the doctor's office were inaccurate, and that they were hopeful the scanner could provide accurate measurement.
Individual	Dealing with language barrier: “I think it is pretty easy for children to understand what we need from them in order for us to get good scans. Challenges only occur when the child is really active …, or when there is some sort of barrier in communication.” Assessing child temperament: “Being able to detect the child's temperament and learning style early on did contribute to the time it took to secure measurements. Each child responded to different distraction techniques and games in various ways. Identifying the type of child being measured early on was often helpful in decreasing measuring time.”	Anthropometrists highlighted individual child characteristics when discussing measurement efficiency and ease of use, referring primarily to child “temperament.” Specific behaviours that made measuring more difficult and time consuming were: being active or unable to stay still, and seeking attention. Distraction techniques had to be adapted to the individual child. Over‐activity and attention seeking were viewed as more problematic for scanning because of the inability to **touch**, and some anthropometrists believed that scanning exacerbated attention seeking. Multiple anthropometrists discussed language as a barrier for efficient scanning because of the inability to communicate positioning to the child or caregiver who acted as an assistant when English was not their first language. Language barriers affected scans and manual measurements because a lack of understanding made the child more afraid.
Child's age	Getting usable scans: “I think the age of the child does have an effect on which measure is faster. Most of the 3 and 4 year olds were easy to scan and measure. Children that were old enough to crawl (about 9 months) and under 3 took longer to scan because we had a hard time keeping them still enough to capture usable 3D images.” Child lacking awareness: “Clearly young infants … are not aware of what is going on … they don't seem to have a reaction to either the scanning or the physical measurements. Children over about 6 months become more difficult to manage. They may not want to stay in the position … for the scanning, and may resist being touched for the physical measurements. Children well over 3 years old frequently do understand … and can be quite cooperative.”	All anthropometrists agreed that the age of the child was the largest determinant of the speed and ease of measuring. Infants under 6 months and children older than 3 years were the easiest to measure. When infants start to turn over and crawl the movement makes measuring more difficult. At 1 year of age awareness increases and children can become “knowingly uncooperative.” Child strength increases with age and children become harder to physically manipulate, which can make measuring more difficult from 1 year of age until the age at which children are better at following directions, 2.5 to 3 years of age. Within the more difficult age group of 6 months to 3 years, children 12–24 months were particularly challenging because they did not like to lie down and are strong enough to resist. While both manual measurement and scanning were more difficult for the middle age group, the inability to touch the child made scanning more difficult for this age group.
Clothing	Undressing a child: “Older children (36 + months) are the age range that generally have the most concern about being undressed. The process of getting the child undressed and into another form of covering has taken up to 10 minutes, multiple visits, assistance from adults the child is comfortable with, and sometimes has required the case to be lost. Regardless of age of the child, parental figures have lost their willingness to consent due to the requirement to undress for scanning.”	All anthropometrists reported that undressing the child was a challenge. Undressing caused distress before measuring began. Anthropometrists related discomfort with undressing to “stranger anxiety.” Older children were more reluctant to undress. One anthropometrist felt that undressing caused children to relate measuring to experience at the doctor's office. Some anthropometrists reported that they themselves felt awkward undressing children, but that it became easier as the study progressed. Anthropometrists, who also recruited for the study, reported that some caregivers were hesitant or refused to consent to the study because children would be undressed.
Experience	Receiving medical care: “The MUAC measuring tape seemed to remind children of the tourniquet applied to the arm before shots are administered.” Receiving medical care: “A pen or marker is typically used to mark the midpoint which can sometimes be confused as a needle for taking a shot and can have adverse effects on the child's behavior.” Comfortable because familiar: “I believe that children are more comfortable with a tape measure and the measuring board. These are items they have seen before and have some understanding of how they work.” Taking a picture: I think the older children actually enjoy doing the scans, because they think they are being photographed doing poses.	All anthropometrists commented that the previous experience of the child affected the measurement experience. One anthropometrist commented that children were taught not to undress for strangers, and another reported that undressing reminded children of visiting the doctor. One anthropometrist felt that children were more comfortable with manual measurement because they were familiar with the equipment. The most commonly reported beliefs from the anthropometrists were that children related scanning to having their picture taken and manual measurement to going to the doctor's office. All anthropometrists said that manual measurement equipment made children relate the experience to going to the doctor, sometimes causing distress and uncooperative behaviour. The children themselves made comments that convinced anthropometrists that they thought it was a doctor visit. Children were familiar with tablets and phones; and all anthropometrists agreed that older children related scanning to taking a picture. For the most part the idea of taking a picture made children more cooperative, but some felt it exacerbated attention seeking in some cases. The scanner made a “clicking” sound, which may have reinforced the idea of taking a picture.
Touch	Holding the child: “Being able to hold the child in order to complete measurements (on the length board for example), certainly makes the process much faster. Not being able to hold the child frequently makes the scan process take much longer …” Holding the child: “While capturing scans it would be extremely helpful if the child could be touched. This would aid in keeping them still and in their proper poses.” Getting frustrated: “… the experience of taking scans with the really uncooperative child is so emotionally infuriating … It's pretty common for it to be difficult to do physical measurements where the kids scream and you can just tune them out, but when you try to take scans and they are running around it's so frustrating and it makes you really upset … [it happened] probably once a day.”	Anthropometrists reported that some children were sensitive to being touched by strangers. For children that were sensitive to touch manual measurement was more distressing to the child than scanning, but anthropometrists did not consider touch sensitivity a big issue. The larger issue with touch was the inability to touch children during scanning, which made positioning the child and keeping the child still much more difficult and time consuming. Through trial and error anthropometrists started to use long spoons—during scanning the child could hold one end while the anthropometrist held the other end of the spoon, and it did not affect the quality of the scan or the ability to process the scan. The use of spoons helped mitigate the impact of not being able to touch the child, but it did not always work; and it was common for scanning to take longer for active children that did not follow instructions. For some anthropometrists the inability to physically restrain children during scanning was a frequent source of frustration. Others reported that feelings of frustration were not so frequent.
Safety	Children playing with equipment: “The board does not move smoothly, so sometimes it can bump a child on the head as it snaps into place. The other main issue with the height board is that children often like to try to measure their own head, grabbing the moving part of the board and pulling it down. If the measurer isn't quick enough, this results in them hitting themselves pretty hard on the head. I don't think I've ever seen a child become upset by this though.”	Anthropometrists did not report any harm to a child from scanning or manual measuring. The only reported safety concerns of the anthropometrists were that moving pieces of manual equipment could potentially hurt children, and anthropometrists did sometimes worry about hurting the child when physically manipulating them into position for manual measurement. For scanning, sanitization of equipment was not necessary, and one anthropometrist mentioned that there was less chance for spreading pathogens during scanning because there was less physical contact. Anthropometrists reported that some caregivers showed concern over 3D scanning “being harmful to the child internally,” and over taking pictures of children without clothing.
Environment	Other children influence cooperation: “A factor to consider for both measuring types is the environment around the children. If it is cold in the room, both processes can be very uncomfortable. Some children do better with other peers around and some do not. Even things like having other toys in the room can be a distraction for both processes and increase the time spent on each case.” Lacking certainty: “I still have not figured out with the lighting how changing it affects the scans because it's not consistent.” Selecting scan location: “[Scanner malfunction from lighting] probably happened at every site when we first got there. At Midtown we were in different rooms every time and we had to figure that out every single time.”	Anthropometrists mentioned some environmental concerns that affected both manual measurements and scanning, such as cold causing children to be uncomfortable and objects or other children in the room affecting cooperation. There were additional environmental factors that were reported only in relation to scanning. A flat surface was necessary for scanning, as was adequate space. Anthropometrists found that they needed enough distance between themselves and the child to capture the entire child in a scan, and that narrow spaces (such as a hallway) would make the scanner malfunction. Lighting was the most commonly mentioned environmental factor, and it seemed to be the hardest factor to account for. Anthropometrists reported that both natural and fluorescent light affected scans. At the end of data collection anthropometrists still did not always understand why light was causing scanner malfunction and could not always predict where lighting was appropriate for scans. For the most part lighting was not viewed as a big problem; anthropometrists would identify an appropriate place to scan children at each location and stay in that location. At one site anthropometrists had to move from room to room and this was the site where lighting presented the biggest problem for scanning.
Dependability	Lighting affects scan: “Overall, I think that the equipment for the physical measurements were more reliable and consistent because we didn't have to worry about external factors such as lighting or space interfering with these measurements.” Lacking certainty: “There were times that my scans had interference that I couldn't determine the source. Was it poor lighting? Reflection from a surrounding material?” Experiencing glitches: “On many occasions the measuring tapes have become damaged, but they are cheap … The scanning equipment is fairly reliable, but there have been times where there are issues with getting the camera to pick up the child or focus … there have been about four occasions where it took several minutes just to get one scan on a child …”	Anthropometrists rated manual equipment as the most dependable because it was sturdy and consistent. With manual equipment there was no concern of external, environmental factors affecting measurement. Anthropometrists reported that measuring tapes frequently broke, but this was easily dealt with by using replacements. Anthropometrists viewed scanners as generally dependable, but there were exceptions. Scanners were viewed as “surprisingly sturdy.” There were no reported instances of 3D scanners getting damaged or breaking. Anthropometrists reported that charging the scanner and iPad was not a big burden and only took an hour, but sometimes operators forgot to charge in the evening and this caused delays in data collection. The main reason scanners were rated less dependable than manual measurement is that they did not always function properly. Anthropometrists reported experiencing “glitches” that caused delays in data collection. Malfunctioning was frequently attributed to lighting and in every location anthropometrists spent time to find a spot with appropriate lighting. In some cases anthropometrists could not determine the cause of scanner malfunction. Anthropometrists also highlighted that the dependability of the scanner was dependent on the child staying still.

*Note*. BINA: Body Imaging for Nutritional Assessment Study.

#### 3.2.1 Streamlined experience

Favourable perception of the 3D imaging system was dominant. The term “streamlined experience” was borrowed from one of the interviewees, who reported that “scanning equipment … makes the process more streamlined.” We combined “streamlined” with “experience” to emphasize that streamlining could be applied to both physical equipment and the measuring experience and also to highlight the importance of previous child experience.

“One of the major benefits … is that the device is extremely light, can fit in a small bag, and is very easy to operate. This is unlike the manual equipment, which is heavy and cumbersome … The scanning equipment also does not need to be sanitized … Further, the scanning equipment can double as … an entertainment device …”

The above quote highlights the physical characteristics of scanning equipment and how the use of a scanner affects other equipment needs. In addition to being smaller and lighter than a length board and a single piece of equipment compared with the multiple tools required for manual measurement, scanning reduced the need to carry additional supplies to sanitize the measuring equipment, and toys or other devices to encourage cooperation from children. Another anthropometrist stated that “the 3D imaging device … eliminates the need for other resources” and referred to an additional advantage of the scanner doubling as an instrument to record measurements.

For an anthropometrist, the experience of measuring begins with learning to use anthropometric tools, and all five anthropometrists commented that learning to take scans was easy, pointing out that “besides having to adjust the box on the screen to fit the object/person … it is just like taking a picture” and that there was “not a lot of user input required to actually perform the scan.” For the most part, the ease of learning to scan carried over to using the scanner in the field.

Most anthropometrists felt that, in general, taking scans was easier and faster than manual measurements. They pointed out that scanning saved time because they did not need to set up and sanitize equipment or record measurements. They also felt that children were less fearful of scans than manual measurement because they related manual measurement to a painful visit to the doctor's office and scanning to “getting their picture taken.” Anthropometrists felt that children were familiar with tablets and taking pictures and that this familiarity made it easy to establish rapport. They also reported that confinement in the length board was a major source of distress for children. Overall, AutoAnthro provided a “streamlined experience”—it was easy to learn, the scanner itself was convenient, children did not experience stress, and taking scans was like taking a few pictures. However, all anthropometrists pointed out that taking scans was not always easy.

“I think that overall, the scanning technology is easier/faster/more convenient for children of all ages. If I were tasked with measuring children with either tool, I would want to have the scanning technology as my primary method, and have the traditional tools as a backup for cases where it wasn't feasible.”

The anthropometrist quoted above preferred scanning over manual measurements but also felt that scanning may not always be feasible. All anthropometrists reported that scanning was “difficult, slower, and less dependable [than physical anthropometry] with an uncooperative/misbehaving child.”

#### 3.2.2 Quality control

Anthropometrists' view of scanning as a streamlined approach changed when capturing a good scan was out of their control. Scanning was dependent on child cooperation because movement affected the ability to capture high‐quality scans, and lack of cooperation was a common issue with children between the ages of 6 months and 3 years.

I would say 3D scanning is more difficult, slower, and less dependable [than manual measurement] with an uncooperative/misbehaving child. Since we can't touch them while getting a scan, they can run or move around, making it impossible to capture a good scan.

The problem of movement during scanning was exacerbated by two factors. First, the best solution to keep an active child still for the required second would be to physically hold them, but this was not possible during scanning because scan processing software required physical separation. Anthropometrists relied on various techniques to foster cooperation, but none of the techniques worked all of the time, and sometimes, anthropometrists “gave up getting good scans and decided to settle for … subpar scans.” Second, anthropometrists were confident in identifying a good or bad scan, but for a scan that was somewhere in between good and bad, a “subpar scan,” they were not certain if the scan was of adequate quality to process into accurate measurements.

Another less common situation when anthropometrists felt that capturing a quality scan was out of their control was when they experienced “software glitches.” Anthropometrists knew that scanners could not function properly in direct sunlight because the scanner relies on infrared light that is washed out by direct sunlight, but they also reported reduced functionality under some “fluorescent lighting.” Dim light did not cause any scanner problems, and for the most part, anthropometrists were able to move around the room and ensure “suitable lighting” through trial and error. This was not a problem when the anthropometrists could stay in one location within a facility for an entire day or multiple days, but it became a challenge when they measured in a hospital setting where they had to move from room to room for each child; they considered finding “suitable lighting” a burden, in part because they could not always predict which lighting conditions constituted “suitable lighting.” On occasion, scanners did not function for an extended period of time, and anthropometrists presumed that light was the likely cause, but they could not identify the exact reason and sometimes referred to such situations as “software glitches.”

The scanning equipment is fairly reliable, but there have been times where there are issues with getting the camera to pick up the child or focus, which is very frustrating … there have been about 4 occasions where it took several minutes just to get one scan.

At times, anthropometrists were uncertain they would be able to complete a set of scans. With manual measurements, there was little concern about completing measurements; uncooperative children could be held, and there were no glitches with manual equipment. Additional qualitative findings on efficiency and invasiveness are integrated into the discussion.

## 4 DISCUSSION

Four out of the five anthropometrists reported that scanning was faster than manual measurement, and quantitative measurement showed that on average, the time required for manual measurements was approximately two times longer than scans. During observation, we found that nearly all crying episodes occurred exclusively during manual measurement, and in interviews, four out of five anthropometrists indicated that children were more comfortable with scans. For the most part, the quantitative and qualitative components of this study were in agreement. Anthropometrist interviews provided further insights into efficiency, invasiveness, and the user experience, including that increased efficiency and reduced invasiveness made scanning a “streamlined approach” for most children, but that scanning was not easy for uncooperative children.

The vast majority of studies and research using 3D imaging for anthropometry did not include children under 5 years of age because imaging systems were not designed to handle movement. To our knowledge, the AutoAnthro System is the first 3D imaging system designed specifically for full‐body anthropometry of infants and young children. The only other 3D imaging system designed for young children is StarScanner (Orthomerica, Orlando, FL), an approved medical device for measuring a newborn's head to design orthoses for cranial remoulding (Ifflaender, Rudiger, Koch, & Burkhardt, 2013). The AutoAnthro and StarScanner systems share the same capture strategy for handling movement—taking multiple scans of short duration and stitching them together. Our study showed that the capture strategy worked well for newborns, infants under 6 months of age, and children 3 years of age and over. However, anthropometrists felt that for up to one half of children 6 months to 3 years of age, it was difficult to get them to stay still long enough for multiple, 1‐s scans and that often they settled for “subpar scans.” Interestingly, in a previous publication, we showed that in BINA, the reliability of scan‐derived measurements was not affected by the age of the child (Conkle et al., 2018), which suggests that many of the “subpar scans” were good enough and cooperation did not often have an effect on measurement quality. BINA anthropometrists took more than six scans and selected the six scans that they considered as the “best quality” for processing into each measurement. We do not know if cooperation affects scan‐derived measurement quality when anthropometrists take only six scans. More research is needed to determine how often scans are of insufficient quality, and AutoAnthro should be improved to give anthropometrists confidence that they are capturing good quality scans. An ideal solution that is simple, but technologically complex, would be to redesign the software to allow the anthropometrist or caregiver to hold the child during scanning. If quality is rarely affected by movement with the current software, as is suggested by reliability data, it may be sufficient to offer improved operator feedback that allows anthropometrists to distinguish between a “subpar scan” and a scan of adequate quality. Information on scan quality could be built into the software or provided through supervision. The need for additional feedback was expressed by an anthropometrist in the quote below.

… we have not had sufficient feedback to know if all our submitted images are adequate. We do get this type of feedback on our manual measurements. We can see if our first and second set of measurements are close and we can compare … to that of our partner … The times that we tested this and found that we were measuring consistently too big or too small, we could correct our technique.

Before BINA, we did not have data on the reliability and accuracy of scan‐derived anthropometry; now that we have such data, it is possible to develop metrics of scan quality in relation to anthropometry.

The current cost of the 3D scanner used in the AutoAnthro System (USD $379; Occipital Inc., 2017) is more than a length/height board (USD $122; United Nations Children's Fund 2017). The requirement to attach the 3D scanner to a cell phone or tablet adds cost to the imaging system, but electronic data capture is becoming more common in clinics and surveys, and the added cost of a mobile device is likely offset by eliminating the need for paper and data entry. In this study, we found that 3D imaging brought efficiency gains related to training, staff, and measurement time that may help to further offset increased costs. We spent 1 day on 3D imaging training, and anthropometrists felt that learning to use the scanner was easy. Our previous findings on reliability suggest that the 1‐day training on 3D imaging was sufficient because scan‐derived measurements were reliable and between‐measurer reliability was the same as within‐measurer reliability (Conkle et al., 2018). Reduced training time could offer savings over traditional anthropometry, which relies on 1‐ to 2‐week trainings of anthropometrists to achieve quality results. In addition to substantial training, manual anthropometry requires the use of a trained assistant. Anthropometrists felt that an assistant was needed for 3D imaging to help position the child, but that “it doesn't necessarily have to be someone who is trained.” In some settings, 3D imaging may be able to rely exclusively on the caregiver to act as an assistant, reducing staff needs. The garment industry saw 3D imaging as a way to reduce the time required to carry out large sample size surveys with up to 40 separate manual measurements of each individual (Jones et al., 1989). We found that 3D imaging took less time than the three manual measurements included in our study. For a household survey, the small difference in measurement time between scans and manual measures may not represent a meaningful difference for efficiency because much more time is spent travelling from house to house. For large‐scale screening, however, saving a minute per child could be considered important. However, in the health sector, only child weight and length/height are commonly measured, with head circumference limited to newborns and infants, and MUAC used as an alternative to weight for length/height. Additional manual measurements are generally not used because of the difficulty and burden of measuring. Compared with the one or two common, manual measurements, 3D imaging does little to reduce measurement time, but it does provide an opportunity to develop novel anthropometric indicators that are not feasible with manual measurement and that may be better predictors of outcomes of interest. Efforts to create new indicators based on 3D measures have already started, with the development of the Body Volume Index and the Health Index (Barnes, 2010; Lin et al., 2004). It is difficult to quantify the future value of new anthropometric indicators. Portability and reduced invasiveness are additional but important advantages of 3D imaging that are difficult to assign value to. The smaller dimensions and reduced weight (<0.5 kg) of the AutoAnthro System lessen the burden on anthropometrists and may reduce transportation costs when compared with a typical, wooden length/height board (7.7 kg; United Nations Children's Fund), and anthropometrists reported that children experienced less stress during 3D imaging. After additional research is carried out and the scanning protocol is finalized, our findings can help to design a comprehensive costing study.

An important strength of this study is the research design; we used a mixed‐methods, collaborative design that increased the relevance and trustworthiness of findings. However, there are some limitations that need to be considered when interpreting or generalizing the findings. The BINA study was designed so that children were scanned first and manually measured second. Because the time‐motion and qualitative components of BINA were carried out in the final weeks of data collection, we chose to maintain consistency and not randomize the order of scanning, which could have biased our findings. We did not consider the time needed to process scans into measurements because the imaging system was designed to be fully automated. However, anthropometrists had to select the 12 best scans when they took extra scans, which was a frequent occurrence, and selection took a substantial amount of time. We expect that an updated version of AutoAnthro will be fully automated, but with the current version, we underestimated scan measurement time by not taking into account selection and deletion of scans. There was also potential to exaggerate the difference between scan and manual measurement times because the BINA protocol included automatic triggers based on reliability for a third manual measurement, but no triggers for a third scan. However, in the time‐motion study, a third measurement was triggered on only one occasion, and it did not meaningfully affect our results. Both qualitative and quantitative findings were based on a small sample size. According to the anthropometrists, scanning took longer for uncooperative children compared with cooperative children. We therefore expected to find a difference in scanning time by age because most uncooperative children were under 2 years of age, but this was not the case; the lack of differences may however be due to the small sample size for the time‐motion study. For the interviews, there were only five anthropometrists with sufficient experience using the AutoAnthro System, and all of them had postsecondary education and were familiar with electronic devices such as tablets and smartphones. Future research on user experience could include multiple FGDs to get input from a larger number of anthropometrists, and findings from this study should not be extrapolated to anthropometrists or children/caregivers with less education and/or limited experience using similar technology. The sensitivity of scanners to light may be more problematic when scanning outside of a building or with frequent movement from house to house, and if caregivers and children lack previous experience with mobile devices, they may react differently to the technology. Anthropometrists reported that some primary caregivers did not consent to their child participating in the study because of privacy concerns and that the safety of the 3D scanner was a common concern of caregivers during recruitment. In BINA, we did not use a formal sampling frame, and it was not possible to determine the percentage of caregivers that refused consent. An additional limitation is that we did not collect qualitative data directly from parents/caretakers or children. Because we conducted most of the measurements in day care and hospital settings, caretakers were not often present during data collection and the majority of children in the study were not old enough to be interviewed.

In a previous publication, we described the need for further research on AutoAnthro to replicate reliability findings, to remove systematic inaccuracy, and to test how well the 3D scanner functions under the harsher conditions of a household survey in a developing country (Conkle et al., 2018). This study further supports the need for additional research before we can make a recommendation for the widespread use of AutoAnthro in surveys or regular nutritional assessment. Specifically, research to develop scan quality control mechanisms is needed. In addition, the scanner needs to be tested in a setting where the general population is not familiar with similar technology and where anthropometrists are not well educated and well trained. As this study showed, it is important that future studies include a qualitative component to provide a comprehensive evaluation. Our findings on efficiency, invasiveness, and the user experience could vary dramatically in a different setting. A household study could determine the likelihood of a caregiver refusing to have their child scanned, and including qualitative research at the household would enable interviewing of caretakers, who may provide valuable insights into invasiveness and the general experience with the technology. Ultimately, to determine the readiness of a 3D imaging system for routine nutritional assessment, the system needs to be tested under normal operating conditions of health facilities and regular surveys.

## 5 CONCLUSIONS

In this study, we found that anthropometrists were not yet ready to completely abandon traditional, manual equipment for 3D scanners. For most children under 5 years of age, 3D imaging was an efficient and non‐invasive way to capture anthropometric data. Revising the AutoAnthro System to address anthropometrists' concerns on capturing good quality scans of uncooperative children should help to facilitate widespread use of 3D imaging for child anthropometry in the health sector.

## DISCLAIMER

The findings and conclusions in this article are those of the authors and do not necessarily represent the official position of the Centers for Disease Control and Prevention.

## CONFLICTS OF INTEREST

All authors do not have affiliations or financial involvement with any organization or entity with a financial interest in the subject matter or materials discussed in the manuscript. Funding sources had no role in data analyses, data interpretation, or report writing. The corresponding author had full access to all data, final responsibility for the decision to submit for publication, and has no conflicts of interest to disclose.

## CONTRIBUTIONS

JC, UR, PS, and RM supervised data collection. KK, AH, and JB, collected manual anthropometry and scans. JC, KK, AH, and RM were responsible for conceptualization and design of the study. JC and AH were responsible for data analysis. JC was responsible for initial interpretation of results and drafting the initial article. All authors contributed to further interpretation of results and critical revision of the article. JC was responsible for article finalization, and all authors approved the final article for publication.

## References

[mcn12686-bib-0001] Adler, C. , Steinbrecher, A. , Jaeschke, L. , Mahler, A. , Boschmann, M. , Jeran, S. , & Pischon, T. (2017). Validity and reliability of total body volume and relative body fat mass from a 3‐dimensional photonic body surface scanner. PLoS One, 12(7), e0180201 10.1371/journal.pone.0180201 28672039 PMC5495384

[mcn12686-bib-0002] Assaf, S. , Kothari, M. , & Pullum, T. (2015). An assessment of the quality of DHS anthropometric data, 2005–2014 In DHS methodological reports no 16. Rockville, MD, USA: ICF International.

[mcn12686-bib-0003] Barnes, R. (2010). The body volume index (BVI): Using 3D scanners to measure and predict obesity. Paper presented at the International Conference on 3D Body Scanning Technologies, Lugano, Switzerland.

[mcn12686-bib-0004] Charmaz, K . (2006). Constructing grounded theory. London; Thousand Oaks, Calif.: Sage Publications.

[mcn12686-bib-0005] Charoensiriwath, S. (2008). A real‐time data monitoring and management system for Thailand 2019 first national sizing survey. Paper presented at the PICMET '08–2008 Portland International Conference on Management of Engineering & Technology.

[mcn12686-bib-0006] Conkle, J. (2017a). Body imaging for nutritional assessment study. Retrieved from http://osf.io/wmbs2 10.1371/journal.pone.0189332 PMC5730209 29240796

[mcn12686-bib-0007] Conkle, J. (2017b). Development and evaluation of a 3D imaging system for child anthropometry. (Ph.D.), Emory University, Atlanta.

[mcn12686-bib-0008] Conkle, J. , Ramakrishnan, U. , Flores‐Ayala, R. , Suchdev, P. S. , & Martorell, R. (2017). Improving the quality of child anthropometry: Manual anthropometry in the Body Imaging for Nutritional Assessment Study (BINA). PLoS One. 10.1371/journal.pone.0189332 PMC5730209 29240796

[mcn12686-bib-0009] Conkle, J. , Suchdev, P. S. , Alexander, E. , Flores‐Ayala, R. , Ramakrishnan, U. , & Martorell, R. (2018). Accuracy and reliability of a low‐cost, handheld 3D imaging system for child anthropometry. PLoS One. 10.1371/journal.pone.0205320 PMC6200231 30356325

[mcn12686-bib-0010] Corsi, D. J. , Perkins, J. M. , & Subramanian, S. V. (2017). Child anthropometry data quality from Demographic and Health Surveys, Multiple Indicator Cluster Surveys, and National Nutrition Surveys in the West Central Africa region: Are we comparing apples and oranges? Global Health Action, 10(1), 1328185 10.1080/16549716.2017.1328185 28641057 PMC5496063

[mcn12686-bib-0011] de Onis, M. , Onyango, A. W. , Van den Broeck, J. , Chumlea, W. C. , & Martorell, R. (2004). Measurement and standardization protocols for anthropometry used in the construction of a new international growth reference. Food and Nutrition Bulletin, 25(1 Suppl), S27–S36. 15069917 10.1177/15648265040251S104

[mcn12686-bib-0012] Garlie, T. N. , Obusek, J. P. , Corner, B. D. , & Zambraski, E. J. (2010). Comparison of body fat estimates using 3D digital laser scans, direct manual anthropometry, and DXA in men. American Journal of Human Biology, 22(5), 695–701. 10.1002/ajhb.21069 20737619

[mcn12686-bib-0013] Ifflaender, S. , Rudiger, M. , Koch, A. , & Burkhardt, W. (2013). Three‐dimensional digital capture of head size in neonates—A method evaluation. PLoS One, 8(4), e61274 10.1371/journal.pone.0061274 23580107 PMC3620274

[mcn12686-bib-0014] Israel, B. A. , Parker, E. A. , Rowe, Z. , Salvatore, A. , Minkler, M. , Lopez, J. , … Halstead, S. (2005). Community‐based participatory research: Lessons learned from the Centers for Children's Environmental Health and Disease Prevention Research. Environmental Health Perspectives, 113(10), 1463–1471. 16203263 10.1289/ehp.7675 PMC1281296

[mcn12686-bib-0015] Jaeschke, L. , Steinbrecher, A. , & Pischon, T. (2015). Measurement of waist and hip circumference with a body surface scanner: Feasibility, validity, reliability, and correlations with markers of the metabolic syndrome. PLoS One, 10(3), e0119430 10.1371/journal.pone.0119430 25749283 PMC4352076

[mcn12686-bib-0016] Jones, P. R. , West, G. M. , Harris, D. H. , & Read, J. B. (1989). The Loughborough anthropometric shadow scanner (LASS). Endeavour, 13(4), 162–168. 2482808 10.1016/s0160-9327(89)80004-3

[mcn12686-bib-0017] Kim, J. Y. , You, J. W. , & Kim, M. S. (2017). South Korean anthropometric data and survey methodology: ‘Size Korea’ project. Ergonomics, 60, 1–11. 10.1080/00140139.2017.1329940 28504058

[mcn12686-bib-0018] Kuehnapfel, A. , Ahnert, P. , Loeffler, M. , Broda, A. , & Scholz, M. (2016). Reliability of 3D laser‐based anthropometry and comparison with classical anthropometry. Scientific Reports, 6, 26672 10.1038/srep26672 27225483 PMC4880916

[mcn12686-bib-0019] Kuehnapfel, A. , Ahnert, P. , Loeffler, M. , & Scholz, M. (2017). Body surface assessment with 3D laser‐based anthropometry: Reliability, validation, and improvement of empirical surface formulae. European Journal of Applied Physiology, 117(2), 371–380. 10.1007/s00421-016-3525-5 28130628 PMC5313586

[mcn12686-bib-0020] Lin, J. D. , Chiou, W. K. , Weng, H. F. , Fang, J. T. , & Liu, T. H. (2004). Application of three‐dimensional body scanner: Observation of prevalence of metabolic syndrome. Clinical Nutrition, 23(6), 1313–1323. 10.1016/j.clnu.2004.04.005 15556253

[mcn12686-bib-0021] Lipman, T. H. , Hench, K. D. , Benyi, T. , Delaune, J. , Gilluly, K. A. , Johnson, L. , … Weber, C. (2004). A multicentre randomised controlled trial of an intervention to improve the accuracy of linear growth measurement. Archives of Disease in Childhood, 89(4), 342–346. 15033843 10.1136/adc.2003.030072 PMC1719855

[mcn12686-bib-0022] Loffler‐Wirth, H. , Willscher, E. , Ahnert, P. , Wirkner, K. , Engel, C. , Loeffler, M. , & Binder, H. (2016). Novel anthropometry based on 3D‐bodyscans applied to a large population based cohort. PLoS One, 11(7), e0159887 10.1371/journal.pone.0159887 27467550 PMC4965021

[mcn12686-bib-0023] Lopetegui, M. , Yen, P. Y. , Lai, A. , Jeffries, J. , Embi, P. , & Payne, P. (2014). Time motion studies in healthcare: What are we talking about? Journal of Biomedical Informatics, 49, 292–299. 10.1016/j.jbi.2014.02.017 24607863 PMC4058370

[mcn12686-bib-0024] O'Brien, B. C. , Harris, I. B. , Beckman, T. J. , Reed, D. A. , & Cook, D. A. (2014). Standards for reporting qualitative research: A synthesis of recommendations. Academic Medicine, 89(9), 1245–1251. 10.1097/ACM.0000000000000388 24979285

[mcn12686-bib-0025] Occipital Inc . (2017). Structure Sensor product details. Retrieved from https://store.structure.io/store?_ga=2.30735295.1074086949.1503324024-1021633441.1503324024

[mcn12686-bib-0026] Pepper, M. R. , Freeland‐Graves, J. H. , Yu, W. , Stanforth, P. R. , Cahill, J. M. , Mahometa, M. , & Xu, B. (2010). Validation of a 3‐dimensional laser body scanner for assessment of waist and hip circumference. Journal of the American College of Nutrition, 29(3), 179–188. 20833990 10.1080/07315724.2010.10719832

[mcn12686-bib-0027] Pullum, T. W. (2008). An assessment of the quality of data on health and nutrition in the DHS Surveys, 1993–2003. In Methodological Reports No. 6 Calverton, MD, USA: Macro International Inc.

[mcn12686-bib-0028] Robinette, K. M. , Daanen, H. , & Paquet, E. (1999). The CAESAR project: A 3‐D surface anthropometry survey. Paper presented at the Second International Conference on 3‐D Digital Imaging and Modeling (Cat. No.PR00062).

[mcn12686-bib-0029] Shulha, L. M. , Whitmore, E. , Cousins, J. B. , Gilbert, N. , & Al Hudib, H. (2015). Evidence based principles to guide collaborative approaches to evaluation: Technical report. Ottawa: Centre for Research on Educational and Community Services, University of Ottawa.

[mcn12686-bib-0030] UNICEF Supply DIvision Innovation Unit . (2017). UNICEF target product profile: Height/length measurement device (s). Copenhagen: UNICEF Supply Division.

[mcn12686-bib-0031] United Nations Children's Fund . Supply catalogue product details. (2017). Retrieved from https://supply.unicef.org/

[mcn12686-bib-0032] USAID . (2016). Anthropometric data in population‐based surveys, meeting report, July 14–15, 2015. Washington, D.C., USA: FHI 360/FANTA.

[mcn12686-bib-0033] Wang, J. , Gallagher, D. , Thornton, J. C. , Yu, W. , Horlick, M. , & Pi‐Sunyer, F. X. (2006). Validation of a 3‐dimensional photonic scanner for the measurement of body volumes, dimensions, and percentage body fat. The American Journal of Clinical Nutrition, 83(4), 809–816. 16600932 10.1093/ajcn/83.4.809 PMC2723741

[mcn12686-bib-0034] Weathers, W. M. , Khechoyan, D. , Wolfswinkel, E. M. , Mohan, K. , Nagy, A. , Bollo, R. J. , … Hollier, L. H. Jr. (2014). A novel quantitative method for evaluating surgical outcomes in craniosynostosis: Pilot analysis for metopic synostosis. Craniomaxillofac Trauma Reconstr, 7(1), 1–8. 10.1055/s-0033-1356758 24624251 PMC3931770

[mcn12686-bib-0035] Wells, J. C. , Treleaven, P. , & Cole, T. J. (2007). BMI compared with 3‐dimensional body shape: The UK National Sizing Survey. The American Journal of Clinical Nutrition, 85(2), 419–425. 17284738 10.1093/ajcn/85.2.419

[mcn12686-bib-0036] Yin, H. , Dai, Y. , Li, H. , Xie, X. , & Ren, H. (2013). The test–re‐test reliability of routine infant anthropometry at primary care hospitals in Chongqing, PR China. Annals of Human Biology, 40(4), 309–317. 10.3109/03014460.2013.775343 23837861

[mcn12686-bib-0037] Zheng, K. , Guo, M. H. , & Hanauer, D. A. (2011). Using the time and motion method to study clinical work processes and workflow: Methodological inconsistencies and a call for standardized research. Journal of the American Medical Informatics Association, 18(5), 704–710. 10.1136/amiajnl-2011-000083 21527407 PMC3168304

